# Pathology of Hepatocellular Carcinoma and Tumor-Bearing Liver Tissue in Association with *hTERT* Promoter Mutation

**DOI:** 10.1155/2023/4313504

**Published:** 2023-08-09

**Authors:** Anne Kristin Fischer, Alexander Semaan, Anna-Lena Wulf, Christian Vokuhl, Diane Goltz, Hans-Peter Fischer

**Affiliations:** ^1^Institute of Pathology, University of Cologne, Kerpener Str. 62 50937, Germany; ^2^Department of General, Visceral, Thoracic and Vascular Surgery, University of Bonn, Venusberg Campus 1, 53127 Bonn, Germany; ^3^Institute of Pathology, University of Bonn, Venusberg Campus 1, 53127 Bonn, Germany; ^4^Institute of Pathology and Hematopathology Hamburg, Fangdieckstraße 75a, 22547 Hamburg, Germany; ^5^Institute of Pathology Troisdorf, Mendener Str. 12, 53840 Troisdorf, Germany

## Abstract

**Background:**

The *hTERT* promoter mutation represents a common and early event in hepatocarcinogenesis, but its linkage to the morphological status of the underlying liver tissue is poorly understood. We analyzed the connection between the histopathological changes in tumor-bearing liver tissue and the occurrence of the *hTERT* promoter mutation in hepatocellular carcinoma (HCC), correlated with clinical data.

**Methods:**

The study cohort comprised 160 histologically confirmed HCC in patients with or without cirrhosis that were investigated for the *hTERT* promoter mutation. We evaluated the frequency of the *hTERT* promoter mutation in patients with HCC with or without cirrhosis and correlated it with potential clinical and histopathological drivers. In particular, we examined tumor-bearing noncirrhotic liver tissue regarding inflammation; the modified histological activity index (mHAI), fibrosis, and steatosis; and its correlation with the frequency of the *hTERT* promoter mutation in HCC. We evaluated overall survival with multivariate Cox regression. Furthermore, we compared hTERT antibody immunohistochemistry and molecular *hTERT* promoter mutation analysis of both HCC and background liver tissue.

**Results:**

The *hTERT* promoter mutation was especially related to HCC in cirrhotic compared with noncirrhotic liver (*p* < 0.001) and independently of cirrhosis in patients ≥ 60 years (*p* = 0.005). Furthermore, the *hTERT* promoter mutation was associated with cirrhosis caused by alcohol toxicity and hepatitis C virus infection. In noncirrhotic liver tissue, the frequency of *hTERT*-promoter-mutated HCC increased with the degree of inflammation and fibrosis. Nevertheless, 25% of the *hTERT*-promoter-mutated HCC developed in normal liver tissue without HCC risk factors. Multivariate Cox regression analysis did not reveal an influence of the *hTERT* promoter mutation in HCC on overall survival at 3, 5, and 16 years. Immunohistochemical analysis with the hTERT antibodies LS-B95 and 2D8 in *hTERT*-promoter-mutated HCC and *hTERT*-wildtype HCC showed a mildly stronger immunoreaction compared with the tumor-bearing liver tissue (LS-B95: *p* < 0.01, 2D8: *p* < 0.01).

**Conclusions:**

Our study reveals a connection between pathological changes in tumor-bearing liver tissue and the *hTERT* promoter mutation in most HCC, even in noncirrhotic liver tissue. Immunohistochemical hTERT antibodies do not discriminate between *hTERT*-promoter-mutated and wildtype HCC.

## 1. Background

Hepatocellular carcinoma (HCC) is the fifth most common malignant tumor and the third leading cause of cancer-related deaths worldwide. In contrast to other entities, the incidence of HCC has continuously risen despite improved treatment options [1]. The etiology of HCC is diverse, with cirrhosis caused by alcoholic liver disease (ALD) and chronic hepatitis C virus (HCV) and hepatitis B virus (HBV) infections as the leading causes of HCC development.

Many HCC harbor specific molecular point mutations (single nucleotide variant [SNV]) in the *hTERT* promotor gene, namely, C228T and C250T, like other malignancies such as glioblastoma multiforme, bladder carcinoma, thyroid carcinoma, and malignant melanoma [2]. This molecular event appears very early in hepatocarcinogenesis, whereas further tumor progression is accompanied by other activating mutations. Specifically, 6% of low-grade dysplastic nodules, 19% of high-grade dysplastic nodules, and 61% of early HCC in the context of cirrhosis harbor *hTERT* hotspot mutations [3, 4]. In contrast, *hTERT* promoter mutations are not observed in cirrhotic and regenerative liver nodules in livers bearing HCC [4, 5].

Telomerase is an RNA-protein complex that operates against the shortening of telomeres by replacing lost telomere sequences. It is composed of the enzyme telomerase reverse transcriptase (TERT); the telomerase RNA component (TERC), which functions as an RNA template; and the stabilizing protein dyskerin. TERT is expressed during embryogenesis, whereas it is silenced in adult tissue except for stem cells. It is inactivated in mature hepatocytes and cholangiocytes. Conditions of parenchymal damage like chronic inflammation, oxidative stress, or toxicity enhance the physiological loss of telomeres, evoke cellular senescence, and suppress cellular regeneration. Missense mutations in the *hTERT* gene also enhance this process and lead to cryptogenic cirrhosis and nodular regenerative hyperplasia [6]. In liver cancer, further telomere shortening, but also reactivation of *hTERT* with higher TERT expression, is observed in many patients with HCC. In conclusion, telomerase reactivation appears to hamper further telomere shortening and enable tumor cells to infinite proliferation [6].

Few studies have analyzed pathological liver tissue changes apart from cirrhosis and their influence on the development of *hTERT*-promoter-mutated HCC. Therefore, we aimed to quantify and qualify the morphologically pathological changes of the HCC-bearing noncirrhotic liver parenchyma and the connection between pathological tissue changes and the *hTERT* promoter mutation in HCC. Furthermore, we evaluated the association between well-known risk factors such as alcoholic liver toxicity and HBV and HCV infection with *hTERT* promoter mutations in relation to the amount of liver damage.

## 2. Methods

### 2.1. Ethics Statement

The experiments were approved by the Institutional Review Board (IRB) of the University Hospital of Bonn (no. 260/16). Informed consent was obtained from all patients from whom tissue samples were included in the study.

### 2.2. Cohort

The cohort consisted of 160 patients with histopathologically confirmed HCC from the archive of the Department of Pathology, University Hospital of Bonn (1999-2013). Tumor evaluation was based on the latest World Health Organization (WHO) classification of digestive system tumors [7] with updated clinical information. All samples were from treatment-naïve patients prior to surgical acquisition. We defined overall survival (OS) as the time from diagnosis to death. The follow-up range was 16 years. Information on HBV, HCV, hepatitis D virus (HDV) infection, and alcoholic liver disease (ALD) was available in all cases. Furthermore, we had information on diabetes and obesity, as well as additional cancer diseases.

### 2.3. Histological Evaluation

We classified and graded HCC samples according to the most recent WHO classification [7]. We histologically evaluated noncirrhotic liver parenchyma for the stage of fibrosis, inflammatory activity according to the modified histological activity index (mHAI) [8], and steatosis as a percent of steatotic hepatocytes.

### 2.4. Tissue Microarray and Immunohistochemical Staining

Using immunohistochemistry, we examined nuclear TERT expression in HCC samples by using tissue microarrays (TMAs). We assembled four tissue samples with a diameter of 0.1 mm from each tumor and two from the normal liver parenchyma, with a minimum distance of 1 cm from the tumor. Each TMA block contained probes from five patients. We analyzed TMAs for the quantity and intensity of nuclear TERT expression using the LS-B95 and 2D8 antibodies (LifeSpan BioSciences Inc.), which are directed against full-length TERT [9]. We analyzed immunohistochemical nuclear TERT expression via the histoanalytic computer software QuPath (version 0.3.0). For further statistical analysis, we modified the results by the *H* score, calculated as follows: the number of weakly reacting nuclei × (the number of moderately positive nuclei × 2) × (the number of strongly positive nuclei × 3) [10, 11]. The *H* score offers a more appropriate evaluation of absolute immunohistochemical antigen expression.

### 2.5. Molecular Analysis

We used Sanger sequencing for the molecular analysis of the *hTERT* promoter mutation. We extracted DNA from paraffin-embedded material, cleaned it, amplified it by polymerase chain reaction (PCR), and then sequenced it by capillary electrophoresis. We manually performed mutation analysis.

### 2.6. Statistical Analysis

The values are expressed as the mean or median with a 95% confidence interval (CI), unless stated otherwise. We compared continuous variables using a *t*-test, whereas we compared categorical variables using the chi-square test or Fisher's exact test. We analyzed survival by using the Kaplan-Meier method and Breslow and Cox regression using univariate and multivariate Cox proportional hazard models in RStudio (RStudio IDE). For survival analysis, we censored a patient when he/she was lost to follow-up. We considered *p* < 0.05 to be statistically significant.

## 3. Results

The cohort comprised 160 tumors (131 men, 29 women). Of the tumors, 93 had developed in cirrhotic liver tissue (Ishak stage 5-6) and 67 had developed in noncirrhotic liver tissue (Ishak stage 0-4). In women, the proportion of HCC in noncirrhotic liver tissue was significantly higher than in men (*p* < 0.04). Most patients were older than 60 years at first diagnosis (mean: 63.9 years for men and 68.8 years for women). Patients with HCC in cirrhotic liver tissue were slightly younger (mean 64.0 years) than patients with HCC in noncirrhotic parenchyma (mean 70.0 years). There were hotspot *hTERT* promoter mutations in 101 HCC cases (63.1%), with an equal sex distribution. In 100 cases, the mutation was C228T; one case harbored the C250T mutation. A few cases had a background of hemochromatosis, PSC, autoimmune hepatitis, and Fanconi anemia. The clinicopathological characteristics are summarized in Table 1.

### 3.1. Molecular Analysis

The *hTERT* promoter mutation was significantly more frequent in HCC arising in cirrhotic liver tissue than in noncirrhotic liver tissue and was independent of sex (*p* < 0.01) (Tables 2 and 3). The *hTERT* promoter mutation was significantly more frequent in patients over 60 years than in patients under 60 years (*p* < 0.01). In general, 78.9% of cases with HCC in cirrhotic liver tissue with underlying chronic illness harbored a *hTERT* promoter mutation, compared with 68.2% of cases without underlying chronic liver disease (*p* = 0.3) (Table 1). There was a significantly higher frequency of the *hTERT* promoter mutation in clinically documented cases of chronic ALD (*p* = 0.04), HBV infection (*p* = 0.81), and HCV infection (*p* = 0.25) compared with cases without underlying liver disease (Table 1). In cirrhotic liver tissue, the frequency of the *hTERT* promoter mutation increased from HCC G1 (50%) to HCC G3 (94%) (*p* = 0.02). In contrast, in noncirrhotic liver tissue, the frequency of the *hTERT* promoter mutation decreased from HCC G1 (50%) to HCC G3 (36.4%).

**Figure 1:**
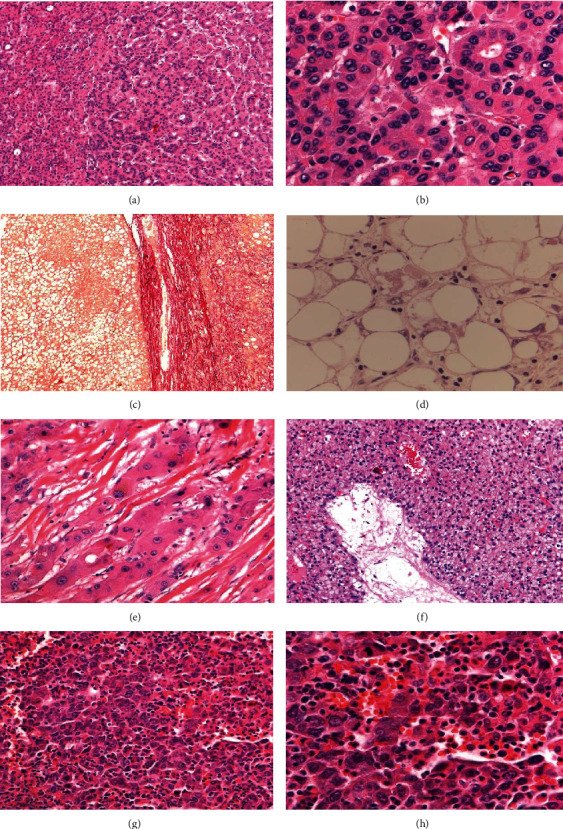
Histological type and grade of the examined hepatocellular carcinoma (HCC): conventional type, pseudoglandular G2 (a, b); steatohepatitic type G1 (c, d); fibrolamellar carcinoma G2 (e); chromophobe type G2 (f); and solid, lymphocyte-rich type G3 (g, h). Except for fibrolamellar carcinoma, all cases have *hTERT* promoter mutation.

We detected the *hTERT* promoter mutation in nearly all histological subtypes of HCC (Figure 1). We observed the highest mutation rates in the solid and steatohepatitic HCC subtype in cirrhotic liver tissue (100% and 88.3%, respectively). Only 62.5% of solid and 33.3% of steatohepatitic carcinomas harbored a *hTERT* promoter mutation in 67 HCC cases in noncirrhotic liver tissue. Tumors in the cirrhotic liver had a significantly smaller mean diameter (3.9 cm in *hTERT*-promoter-mutated and in *hTERT*-wildtype HCC) than tumors in noncirrhotic liver tissue (6.2 cm in *hTERT*-promoter-mutated and 7.7 cm in *hTERT*-wildtype HCC) (*p* < 0.05).

Our main interests were the analysis of HCC arising in noncirrhotic liver tissue with minor histopathological damage and the frequency of *hTERT* promoter mutation in these tumors. Our cohort did not include HCC that developed from a hepatocellular adenoma. Twenty-nine cases had a history of ALD, HBV, or HCV infection, or a combination of these conditions. In strong contrast to HCC in cirrhotic liver tissue, only 41.4% of these tumors harbored *hTERT* promoter mutations (compared with 78.9% of HCC in cirrhotic liver tissue harboring *hTERT* promoter mutations, *p* < 0.01). Furthermore, 47.4% of 38 tumors lacking any clinical risk factors for HCC had an *hTERT* promoter mutation. Moreover, 8 of 18 tumors that arose in noncirrhotic liver tissue without histopathological changes were mutated.

Fifty percent of all tumors in noncirrhotic liver tissue with moderate fibrosis (stages 2-4), moderate inflammation (mHAI > 3), and steatosis (>10%) harbored *hTERT* promoter mutations (Table 3). The frequency of *hTERT* promoter mutations was 30% in tumors that developed in virtually nonaffected liver tissue without fibrosis (stage 0), without or with only sparse inflammation (mHAI 0/1), and without steatosis. This difference, although not significant, reveals an increasing *hTERT* promoter mutation rate associated with chronically affected liver parenchyma, even in noncirrhotic liver tissue. Remarkably, there was a small subgroup of poorly differentiated HCC (grade 3) in noncirrhotic liver parenchyma without any histopathological changes (*n* = 7) and without a *hTERT* promoter mutation.

**Figure 2:**
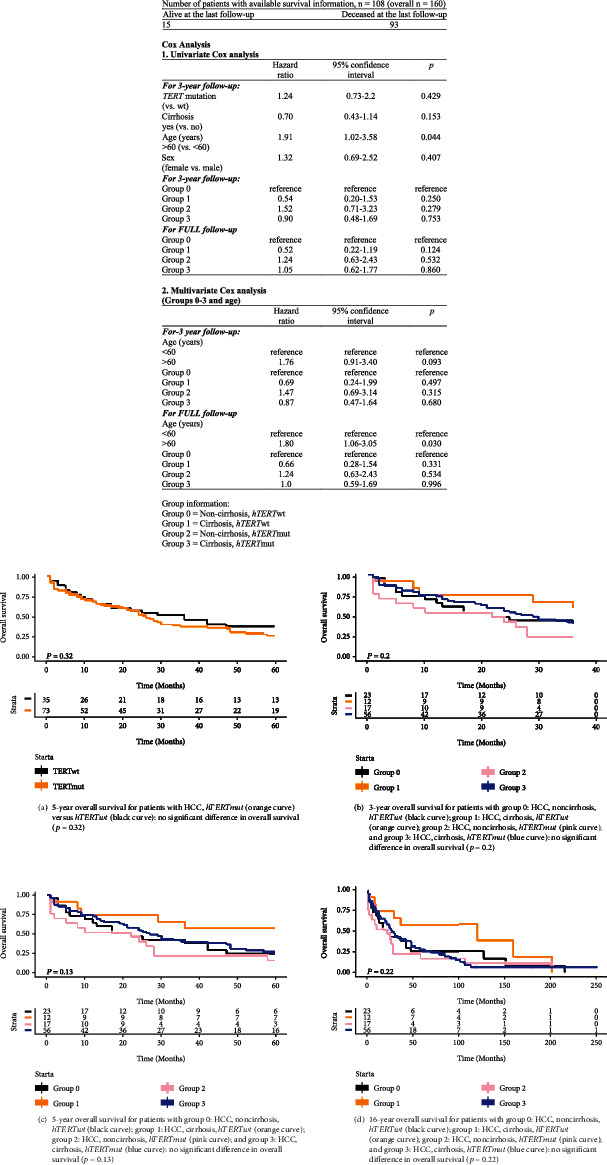
Survival of *hTERT*-wildtype (*hTERT*wt) and *hTERT*-promoter-mutated (*hTERT*mut) hepatocellular carcinoma (HCC), independent of the background liver tissue (a), and of *hTERT*wt and *hTERT*mut HCC in cirrhotic and noncirrhotic liver tissue. Based on age-matched Cox regression analysis (1 and 2), patients with *hTERT*mut HCC in cirrhotic and noncirrhotic liver tissue do not show a significant difference in overall survival at 3 years (b), 5 years (c), and 16 years (d).

To evaluate survival, we performed an age-matched multivariate Cox regression analysis (patients < 60 years vs. ≥60 years) (Figure 2). Over a 5-year period, patients with *hTERT*-promoter-mutated HCC generally had a similar outcome as patients with *hTERT*-wildtype HCC (*p* = 0.32). In a second step, we divided our HCC cohort into four subgroups (Figure 2): subgroup 0: non-cirrhosis, *hTERT* wildtype; subgroup 1: cirrhosis, *hTERT* wildtype; subgroup 2: non-cirrhosis, *hTERT* mutated; subgroup 3: cirrhosis, *hTERT* mutated. There were no significant differences in age-correlated cumulative OS over 3 years (*p* = 0.2), 5 years (*p* = 0.13), and 16 years (*p* = 0.22).

### 3.2. Immunohistochemical hTERT Expression in HCC and Tumor-Bearing Liver Samples

**Figure 3:**
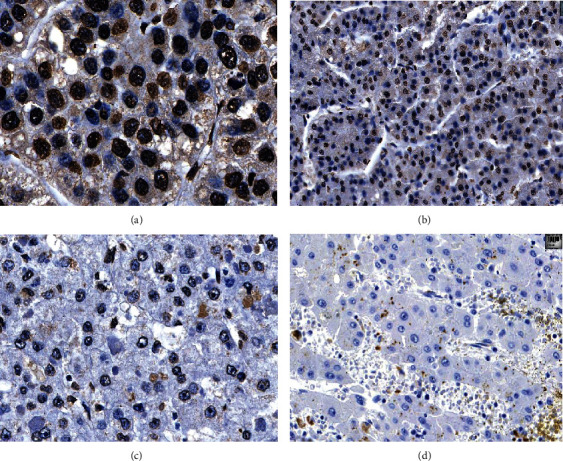
Staining intensity of hTERT antibodies in hepatocellular carcinoma (HCC): strong nuclear staining in a trabecular HCC G2, antibody 2D8 (a). Heterogeneous nuclear staining in HCC G2, antibody LS-B95 (b). Week staining of nuclear membrane in HCC G1, antibody 2D8 (c). Negative staining result in HCC G1, antibody LS-B95 (d).

We also analyzed nuclear hTERT antibody expression in HCC with and without *hTERT* hotspot promoter mutations, as well as in tumor-bearing normal liver samples. We observed either a homogenous or a heterogenous immunohistochemical reaction pattern. In most cases, only a few tumor cells showed strong nuclear staining, whereas the majority had moderate or weak nuclear positivity in the same tissue fragment (Figure 3). Both antibodies showed a similar labeling pattern in HCC and in tumor-bearing liver samples, with significantly stronger expression and higher nuclear staining intensity in tumor tissue (LS-B95: mean *H* score in tumor 106.4, mean *H* score in nontumorous liver 75.9, *p* < 0.01; 2D8: mean *H* score in tumor 103, mean *H* score in nontumorous liver 79.5, *p* < 0.01, *t*-test for independent samples). Comparison of *hTERT*-promoter-mutated HCC and *hTERT*-wildtype HCC revealed a mildly stronger immunoreaction in the first group, but these differences were not significant (LS-B95: *p* = 0.28; 2D8: *p* = 0.16). The hTERT expression was similar in cirrhotic and noncirrhotic liver samples, independent of the presence of a molecularly proved *hTERT* promoter mutation.

## 4. Discussion

HCC development strongly depends on the extent of background liver fibrosis and underlying chronic liver disease. In the recent decade, new molecular genetic aspects have provided additional insights into the pathogenetic connection between different chronic liver diseases and HCC subtypes [6, 12–22]. However, the contribution of nonpathological or only mildly affected background liver tissue on hepatocarcinogenesis remains unclear. We focused on the role of the *hTERT* promoter mutation in HCC and undertook a detailed analysis of the histopathological changes of tumor-bearing liver tissue and the underlying clinically defined liver diseases.

Genome-wide association studies (GWAS) have identified germline and somatic variants in genes that are linked to increased risk for the development of specific chronic liver diseases, especially cirrhosis. The *hTERT* promoter mutations have been revealed as the most common cancerogenic driver mutations in HCC and an early if not the first event in hepatocarcinogenesis. Specific point mutations of the *hTERT* promotor genes (SNV), mostly C228T and rarely C250T, have been reported in well-differentiated nodules of multinodular HCC; additional mutations have only been detected in poorly differentiated progredient tumor nodules [23]. Furthermore, the *hTERT* promoter mutation appears to be the first molecular step in the malignant transformation of *β*-catenin-mutated hepatocellular adenomas [3, 24]. Underlining the noncanonical function of *TERT* in regulating the WNT/*β*-catenin signaling pathway, Trépo et al. [15] found *WNT3A-WNT9A* as a susceptibility locus for alcohol-related HCC, suggesting an early role of the WNT/*β*-catenin signaling pathway in alcohol-related HCC carcinogenesis.

Prior to our study, the *hTERT* promoter mutations had not been detected in nondysplastic cirrhotic liver nodules or in noncirrhotic tumor-harboring liver tissue. Most low- and high-grade dysplastic liver nodules, characterized by histomorphology, did not display *hTERT* promoter mutations [4, 24]. In an East Asian cohort, dysplastic nodules completely lacked *hTERT* promoter mutations [5]. It seems that this molecular event mostly occurs during the malignant transformation of a cirrhotic liver nodule. Considering these conflicting observations, we systematically analyzed possible correlations between histopathological changes in tumor-bearing noncirrhotic and cirrhotic liver tissue and the incidence of *hTERT* promoter mutations in HCC.

We confirmed a high rate of *hTERT*-promoter-mutated HCC in cirrhotic liver tissue (76.3%), a finding similar to another European collective (64%) [24]. Both European cohorts showed a much higher incidence than the East Asian cohorts of Lee et al. [5] (43.1%), Chen et al. [25] (48.2%), and Yang et al. [26] (31.8%). Putatively, these geographical differences might be influenced by genetic factors. Furthermore, the patient's age had an important impact on the occurrence of *hTERT* promoter mutations in HCC. We found the highest rate of *hTERT*-promoter-mutated HCC (83.3%) in patients with cirrhosis and older than 60 years. In this group, the physiological aging of the liver together with accelerated aging due to chronic liver disease might cause a higher consumption of telomeres, a reactivation of telomerase, and presumably an increase in the risk of an activating *hTERT* promoter mutation [27].

We also focused on *hTERT*-promoter-mutated HCC in noncirrhotic liver. Under these conditions, the frequency of *hTERT* promoter mutations was significantly lower than in HCC in cirrhotic liver tissue (44.8% vs. 76.3%). Nevertheless, the occurrence of *hTERT* promoter mutations is still much higher than in many other common cancers like carcinomas of the breast, prostate, colorectum, and lung [2]. Even in noncirrhotic liver, the *hTERT* promoter mutation in HCC still represents a predominant molecular alteration, like in some of the abovementioned tumors [2, 28]. Based on data from a Taiwanese cohort, the incidence of HCC is age-related in cirrhotic and noncirrhotic liver tissue [25]. Furthermore, the rate of *hTERT*-promoter-mutated HCC in our cohort tended to be higher in noncirrhotic liver fibrosis Ishak stage F2-4 and/or mildly active hepatitis with mHAI > 3 and/or steatosis > 10%, compared with HCC in liver tissue without any pathological changes according to Ishak stage F0, mHAI = 0/1, and no steatosis (0%). These findings suggest that the molecular pathogenesis of HCC in the context of the *hTERT* promoter mutation seems to be influenced by chronic histopathological changes of the background liver parenchyma, even in noncirrhotic stages of liver disease. However, some *hTERT*-promoter-mutated HCC, even G3 tumors without aspects of hepatocellular adenoma, arose in completely normal liver tissue. Apparently, chronic liver disease is not an obligatory precondition for the *hTERT* promoter mutation in *de novo* HCC.

Several studies have established a linkage between *hTERT*-promoter-mutated HCC and the etiology of the underlying liver disease. Notably, this connection appears to exist for chronic hepatitis C [5, 28–32] and ALD [30]. ALD or a positive history of chronic alcohol abuse represents the predominant primary liver disease in our cohort of *hTERT*-promoter-mutated HCC with underlying cirrhosis. In contrast, the influence of well-established etiological risk factors for HCC was much lower in our cohort of HCC in noncirrhotic liver. Remarkably, in the subcohort of patients without well-established clinical risk factors and without the relevant, above-mentioned histopathological changes in liver tissue, *hTERT*-promoter-mutated HCC even prevailed over *hTERT*-promoter-mutated tumors with known risk factors. Based on this interesting finding, we suppose that yet unknown factors have to be considered as cause of the *hTERT* promoter mutation in HCC.

The *hTERT* promoter mutation seems to influence the grade and subtype of HCC in cirrhotic and noncirrhotic liver tissue: HCC G3 in cirrhotic liver harbors a significantly higher frequency of the *hTERT* promoter mutation than HCC G1 (*p* = 0.02). Furthermore, HCC G3 in cirrhotic liver has a significantly higher frequency of *hTERT* mutation than HCC G3 in noncirrhotic liver tissue (*p* = 0.02). Remarkably, we found a high frequency of *hTERT*-promoter-mutated steatohepatitic HCC in the cirrhotic liver (88.3%), which even exceeded the mutation frequency in the conventional type of HCC in cirrhotic liver tissue (75.6%). This finding contrasts with the observation of Calderaro et al. [33]. In addition, solid HCC in cirrhotic and noncirrhotic liver tissue showed a higher rate of *hTERT* promoter mutation compared with the conventional type of HCC.

The clinical course and long-term survival analysis over 3, 5, and 16 years revealed no statistical differences in OS for patients with *hTERT*-promoter-mutated HCC and *hTERT*-wildtype HCC in the setting of cirrhosis and non-cirrhosis. These observations concur with the OS analysis of Lee et al. [5] and Chen et al. [25], who focused on HCC under HCV infection.

Immunohistochemical antibodies LS-B95 and 2D8 against TERT did not discriminate between *hTERT*-promoter-mutated and *hTERT*-wildtype HCC. This mirrors the results of other studies [24, 26], and we conclude that the antibodies we evaluated are not appropriate for immunohistochemical analysis to detect the *hTERT* promoter mutation. Molecular analysis remains the method of choice. However, both antibodies revealed significantly enhanced staining in tumor cell nuclei compared with normal hepatocellular nuclei. Telomerase appears to be enriched in some strongly immunoreactive HCC compared with normal liver tissue.

## 5. Conclusion

In conclusion, we revealed the close relationship between cirrhosis and the *hTERT* promoter mutation in HCC. We also elaborated on a putative linkage of the *hTERT* promoter mutation in HCC even in noncirrhotic fibrosis, mildly active hepatitis, and steatosis of different etiologies. This underlines the clinical necessity to repress chronic liver disease even in its early stages. A small subcohort of *hTERT*-promoter-mutated HCC, including G3 tumors without known liver disease and without relevant histopathologic changes of tumor-bearing liver tissue, indicates that currently unknown pathogenetic mechanisms seem to be responsible for the *hTERT* promoter mutation in HCC.

## Figures and Tables

**Table 1:** *hTERT* promoter mutation in hepatocellular carcinoma (HCC): association with causative clinical disease.

	Causative disease		*hTERT* mutation absolute numbers and %	Wildtype *hTERT* absolute numbers and %	Comparative test	*p*
1	Without causative disease	Cirrhosis	15 (68.2)	7		
2	With causative disease	Cirrhosis	56 (78.9)	15	2 vs. 1	0.30^∗^
3	ALD	Cirrhosis	27 (87.1)	4	3 vs. 1	0.09^∗∗^
4	Hepatitis B	Cirrhosis	8 (57.1)	6	4 vs. 1	0.50^∗^
5	Hepatitis C	Cirrhosis	4	13	5 vs. 1	0.57^∗∗^
6	Hepatitis B and C	Cirrhosis	0	1		
7	ALD and hepatitis B	Cirrhosis	1	2		
8	ALD and hepatitis C	Cirrhosis	0	2		
9	ALD in other combinations	Cirrhosis	0	2		
10	Other	Cirrhosis	0	1		
	Total	Cirrhosis	71	22		
11	Without primary disease	Non-cirrhosis	18 (47.7)	20	11 vs. 1	0.11^∗^
12	With primary disease	Non-cirrhosis	12 (41.4)	17	12 vs. 2	<0.00^∗^
13	ALD	Non-cirrhosis	7 (40.0)	6		
14	Hepatitis B	Non-cirrhosis	3 (40.0)	2		
15	Hepatitis C	Non-cirrhosis	3 (50.0)	3		
16	Hepatitis B and C	Non-cirrhosis	0	0		
17	ALD and hepatitis B	Non-cirrhosis	1	0		
18	ALD and hepatitis C	Non-cirrhosis	1	0		
19	ALD and hepatitis B and C	Non-cirrhosis	1	0		
20	Other	Non-cirrhosis	1 (50.0)	1		
21	Total	Non-cirrhosis	30	37		

ALD: alcoholic liver disease. ^∗^Chi-square test. ^∗∗^Fisher's exact test.

**Table 2:** *hTERT* promoter mutation in hepatocellular carcinoma (HCC): general data and association with clinical and pathomorphological findings.

		Number of cases with cirrhosis (absolute numbers and %)	Number of cases without cirrhosis (absolute numbers and %)	Total number (%)	Comparative test	*p*
1	Frequency of cirrhosis	93 (58.1)	67 (41.9)	160		
2	*hTERT* promoter mutation	71 (70.3)	30 (29.7)	101 (63.1)	2 vs. 3	<0.00^∗^
3	Wildtype *hTERT*	22 (37.3)	37 (62.7)	59 (36.9)		
4	Males with *hTERT* promoter mutation	61 (73.5)	22 (26.5)	83 (63.4)		
5	Females with *hTERT* promoter mutation	10 (55.6)	8 (44.4)	18 (62.1)		
6	Patients < 60 years with *hTERT* promoter mutation	14 (56.0)	3 (25.0)	17 (45.9)	6 vs. 7	0.01^∗^
7	Patients > 60 years with *hTERT* promoter mutation	57 (83.3)	27 (49.1)	84 (68.3)		
8	Alcoholic liver disease	31 (71.5)	13 (29.5)	44 (27.5)		
9	Hepatitis C virus infection	17 (73.9)	6 (26.1)	23 (14.3)		
10	Hepatitis B virus infection	14 (76.5)	5 (50.0)	19 (11.9)		
11	G1 HCC with *hTERT* promoter mutation	6 (50.0)	4 (50.0)	10 (50.0)	11 vs. 13 cirrhosis	0.02^∗∗^
12	G2 HCC with *hTERT* promoter mutation	48 (76.2)	22 (45.8)	70 (63.1)		
13	G3 HCC with *hTERT* promoter mutation	17 (94.4)	4 (36.4)	21 (72.4)		
14	Conventional type with *hTERT* promoter mutation	62 (75.6)	12 (50.0)	84 (71.2)		
15	Solid type with *hTERT* promoter mutation	3 (100)	10 (62.5)	13 (68.4)		
16	Steatohepatitic type with *hTERT* promoter mutation	5 (88.3)	5 (50.0)	10 (62.5)		
17	Tumor size (cm); *hTERT* promoter mutation (cm)	3.9 cm	6.2 cm		17 cirrhosis vs. non-cirrhosis	0.01^∗^
18	Tumor size (cm) wildtype (cm)	3.9 cm	7.7 cm		18 cirrhosis vs. non-cirrhosis	<0.00^∗^

^∗^Chi square test. ^∗∗^Fisher's exact test.

**Table 3:** *hTERT* promoter mutation in hepatocellular carcinoma (HCC) in noncirrhotic liver: correlation of mutation frequency with minor histological parenchymal damage (inflammation, fibrosis, and steatosis).

Pathological tissue changes	Number of cases with *hTERT* promoter mutation/number of cases with *hTERT* wildtype	*hTERT* promoter mutation %	Comparative test	*p*
Cirrhosis	71/22	76.3		
Non-cirrhosis, all cases	30/37	44.8	2 vs. 1	0.00^∗^
Non-cirrhosis F = 2-4; mHAI > 3; S > 10	14/14	50		
Non-cirrhosis F = 0.1; mHAI ≤ 3; S ≤ 10	16/23	41	4 vs. 3	0.47^∗^
Non-cirrhosis F = 0; mHAI = 0.1; S = 0	3/7	30	5 vs. 3	0.46^∗^

F: stage of fibrosis [8], with a maximum of 6 points possible; mHAI: modified histological activity index [8], with a maximum of 18 points possible; S: steatosis (in %). ^∗^Chi-square test.

## Data Availability

The data used to support the findings of this study are available from the corresponding author upon request.

## Abbreviations

ALD: Alcoholic liver disease
CI: Confidence interval
DNA: Deoxyribonucleic acid
GWAS: Genome-wide association study
HBV: Hepatitis B virus
HCC: Hepatocellular carcinoma
HCV: Hepatitis C virus
HDV: Hepatitis D virus
mHAI: Modified histological activity index
OS: Overall survival
PCR: Polymerase chain reaction
PSC: Primary sclerosing cholangitis
RNA: Ribonucleic acid
SNV: Specific nucleotide variant
TERC: Telomerase RNA component
TERT: Telomerase reverse transcriptase
TMA: Tissue microarray
WHO: World Health Organization.
